# Susceptibility to Bismuth(III) of Aquaculture Bacterial Pathogens: Effectiveness of Bismuth–Deferiprone Therapy against *Vibrio anguillarum* Infection in Fish

**DOI:** 10.3390/microorganisms9112399

**Published:** 2021-11-21

**Authors:** Miguel Balado, Diego Rey-Varela, Ana M. Albela, Manuel L. Lemos

**Affiliations:** Department of Microbiology and Parasitology, Institute of Aquaculture, Universidade de Santiago de Compostela, 15782 Santiago de Compostela, Spain; diego.rey@usc.es (D.R.-V.); anamaria.albela@rai.usc.es (A.M.A.)

**Keywords:** aquaculture, bacterial pathogens, vibriosis, *Vibrio anguillarum*, bismuth

## Abstract

Bismuth is a heavy metal with antibacterial properties that has a long history of medicinal use. The results reported here suggest that bismuth(III) (chelated with deferiprone) could be used in aquaculture systems to treat bacterial disease outbreaks, greatly reducing antibiotic use. We tested bismuth susceptibility in a collection of aquaculture bacterial pathogens. In the presence of bismuth concentrations ranging from 1.3 to 13 µM, most bacteria started showing a drastic decrease in their growth ability, although with high inter- and intraspecific variability. The minimal inhibitory concentrations of bismuth ranged from 13 to more than 780 µM, depending on bacterial species and strain. The results of in vivo assays suggest that low concentrations of bismuth could be especially effective to treat vibriosis caused by *Vibrio anguillarum,* since bismuth greatly reduced mortality in experimentally infected fish without any observable side effects. A bismuth therapy, alone or combined with other antimicrobials, could contribute to reduce the use of antibiotics in aquaculture.

## 1. Introduction

Aquaculture is one of the fastest growing food sectors in the world and it is rapidly replacing fisheries as source of dietary protein [1]. However, infectious diseases are a major threat to aquaculture production and continuous efforts must be conducted to prevent mortality caused by pathogenic microorganisms. Among bacterial diseases, furunculosis by *Aeromonas salmonicida,* photobacteriosis by *Photobacterium damselae* subsp. *piscicida,* or vibriosis by *Vibrio anguillarum* are three of the most devastating diseases affecting farmed fish worldwide [2]. Although vaccines have proven to be the most effective solution to prevent the occurrence of infectious diseases [3], sometimes, when an outbreak occurs, the application of antibacterial agents is the only alternative to control an acute bacterial disease. However, a global challenge is the appearance of resistance to antimicrobial agents in bacterial pathogens, which increases the interest in metal-based antimicrobials [4,5].

Bismuth is a heavy metal with antibacterial properties that has a long history of medicinal use [6]. Nowadays, bismuth compounds are used in human medicine to control bacteria in gut-related diseases where sulfate-reducing bacteria are implicated [7,8]. It is commonly used in the treatment of stomach ulcers caused by *Helicobacter pylori*. In this case, oral administration of bismuth salts combined with antibiotics has been shown as a highly effective therapy [9]. This therapy strategy reduced development of resistance to coadministered antibiotics [10] and also was effective to control *H. pylori* strains with multidrug resistance [11,12]. Bismuth has been also proposed as a chemotherapic agent against *Pseudomonas aeruginosa*, *Staphylococcus aureus*, *Clostridium difficile,* or even against norovirus [6,13]. The antibacterial properties of bismuth are based on the inactivation of cysteine-rich key proteins of bacterial cells. Bi^3+^ ions have a high affinity for thiolate sulfur and to nitrogen or oxygene ligands [6].

In the present work, we tested the bismuth susceptibility in some strains of aquaculture bacterial pathogens including *V. anguillarum*, *A. salmonicida,* and *P. damselae* subsp. *piscicida*. The results of in vitro and in vivo assays suggest that the use of bismuth chloride at low concentrations could be an effective therapy against certain bacterial infections, such as vibriosis due to *V. anguillarum*, to reduce mortality of disease outbreaks in fish.

## 2. Materials and Methods

### 2.1. Bacterial Strains and Routine Growth Conditions

Bacteria used in the study are listed in Table 1. Bacteria were routinely grown in Tryptic Soy Broth or Agar (Pronadisa, Madrid, Spain) supplemented with 1% NaCl (TSB-1 or TSA-1 respectively) at 25 °C. All strains used in this work were maintained into vials of TSB-1 with 15% glycerol and stored at −80 °C. A fresh culture was prepared from these samples before each assay.

### 2.2. Preparation of Bismuth Stock Solution

Bismuth stock solution was prepared by chelating it with deferiprone (3-hydroxy-1,2-dimethyl-4(1H)-pyridone) (Fisher, Waltham, MA, USA). Deferiprone:bismuth at a molar ratio of 5:1 was prepared by adjusting a solution of 0.1 M deferiprone to pH 3.0 and adding 0.02 M BiCl_3_ (Fisher) to the chelate solution. After bismuth was chelated by deferiprone, the pH of the colorless solution was adjusted to 7.2 with NaOH 1 M [14]. The final concentration of bismuth was 10 mM.

### 2.3. Test for Inhibition of Bacterial Growth

To determine the susceptibility of tested bacteria to bismuth, bacterial strains were cultivated in TSB-1 under increasing concentrations of bismuth chloride. Growth assays were performed in 96-well microplates containing 200 µL of medium in each well. Overnight cultures of each bacterial strain were adjusted at OD_600_ = 0.5 and diluted 1/20 in fresh TSB-1 medium containing bismuth chloride at increasing concentrations (between 0.13 µM and 1 mM) to calculate the minimum inhibitory concentration (MIC) for each strain. Microplates were incubated at 25 °C for 16 h and the growth achieved was measured with a spectrophotometer at 600 nm. The MIC concentration was the lower Bi concentration at which no growth was observed. To compare susceptibility at different Bi concentrations, the ratio (percentage) between OD_600_ achieved at a concrete Bi concentration and that observed in control without bismuth was calculated. Each growth measurement was performed in triplicate.

### 2.4. Evaluation of Bismuth–Deferiprone against V. anguillarum in Experimental Infections

Experimental infections using Senegalese sole (*Solea senegalensis*) juveniles were used to evaluate the bismuth antibacterial therapy to treat fish vibriosis. To carry out experimental infections, 100 fish with an average weight of 100 g were randomly divided into four groups of 25 animals. Each fish group was maintained in 50-L seawater tanks at 17 °C with continuous aeration. Three groups (75 fish) were inoculated intraperitoneally (IP) with 0.1 mL of a bacterial suspension at 3–5 × 10^5^ colony-forming units (CFU) per mL in saline solution (0.85% NaCl). This suspension was obtained by 10-fold serial dilutions of a bacterial suspension at an OD_600_ = 0.5 prepared by suspending several colonies of *V. anguillarum* RV22 from a 24 h TSA-1 culture. The precise number of injected bacterial cells was determined by plate count of 10-fold serial dilutions on TSA-1. Four days after the pathogen inoculation, infected fish from the three groups were again randomly mixed and subjected to one of the following treatments by IP injection (0.1 mL): The first group was treated with saline solution; the second group was treated with a solution of bismuth chloride at 1.3 µM; and the third group with bismuth chloride at 13 µM. A fourth control group of 25 animals was treated first with saline solution instead of bacterial suspension and on day 4 was injected with 0.1 mL of bismuth at 13 µM. In addition, a fifth group of 25 animals was used as “manipulation control” since the fish were injected both times with saline solution and subjected to the same manipulation as the rest of groups. Mortalities were recorded daily for 10 days after injection and statistical significance of differences in survival functions were determined using the Kaplan-Meier method with Mantel-Cox log-rank test using SPSS (version 20; IBM SPSS Inc., Chicago, IL, USA). *p* values were considered significant when *p* was < 0.05. The protocol for animal experimentation used in this study has been reviewed and approved by the Animal Ethics Committee of the University of Santiago de Compostela (Protocol approval number: 15004/2015/002).

## 3. Results

### 3.1. Susceptibility to Bismuth of a Collection of Aquaculture Bacterial Pathogens

**Figure 1:**
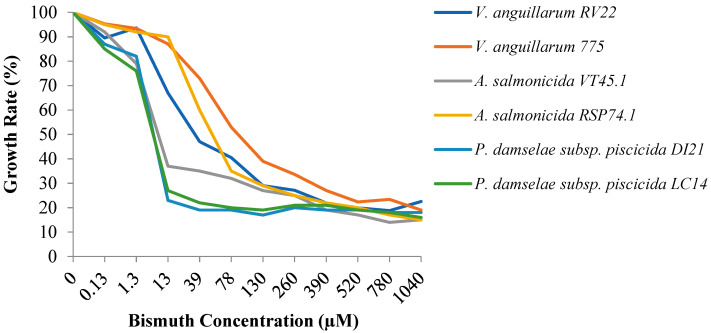
Maximum growth achieved by two strains of the three main fish pathogens studied, when they were grown in Tryptic Soy Broth with 1% NaCl (TSB-1) containing bismuth–deferiprone. Growth values shown are the ratio of the growth achieved in TSB-1 at each bismuth concentration divided by growth in TSB-1 without bismuth.

To evaluate the usefulness of bismuth therapy to treat bacterial infections in aquaculture, three main fish pathogens, *Vibrio anguillarum*, *Aeromonas salmonicida,* and *Photobacterium damselae* subsp. *piscicida* were first assayed for in vitro bismuth susceptibility assays. The growth in presence of different concentrations of Bi(III) showed a general tendency to decrease when the concentration of the metal increased, even at low concentrations. In presence of low concentrations of bismuth, ranging from 1.3 to 13 µM, most bacteria showed a drastic decrease in their growth ability. *P. damselae* subsp. *piscicida* showed the greatest susceptibility since its growth capacity was greatly reduced with the addition of 13 µM Bi(III) (Figure 1). The addition of 13 µM also reduced the growth of *A. salmonicida* RSP74.1. Interestingly, *A. salmonicida* VT45.1 displayed at this concentration a growth of 70% with respect to the medium without Bi(III), being necessary 78 µM to inhibit it. Among the three main fish pathogens tested, *V. anguillarum* showed the lowest susceptibility to bismuth. A Bi(III) concentration of 130 µM was necessary to reduce growth by 50% of *V. anguillarum* 775, and 39 µM for *V. anguillarum* RV22.

In addition, the minimal inhibitory concentration (MIC) of Bi(III) was also determined in a collection of pathogenic bacteria, mainly Vibrios, isolated from diseased fish or mollusks (Table 1). In most strains tested, a growth reduction began at Bi(III) concentrations between 13 and 130 μM. *A. salmonicida*, *V. costicola,* and *V. fischeri* showed the greatest susceptibility to Bismuth, since their growth was significantly reduced at a concentration of 1.3 µM, and their MIC was 13 µM. A Bi(III) concentration of 39 μM was necessary to start reducing growth of most tested species, including *A. hydrophila, V. diazotrophicus, L. garvieae, L. piscium,* and *V. campbellii*, although MICs showed diverse values (Table 1). The *Vibrio* species less susceptible were *V. alginolyticus*, *V. campbellii*, *V. diazotrophicus*, *V. furnissii,* or *V. metschnikovii*, which showed MICs of more than 780 µM. In contrast, in the range of bismuth concentrations used, only a slight reduction in the growth of *Y. ruckeri* was observed, suggesting a much less efficient inhibition compared to the genus *Vibrio*. The results show that a Bi(III) concentration ranging between 13 and 130 μM can potentially reduce the growth of most fish pathogenic bacteria. Overall, the results showed that there are important differences in susceptibility to bismuth not only interspecific but also intraspecific, between strains of the same species.

### 3.2. Effect of Siderophore Production to Bismuth Susceptibility

**Figure 2:**
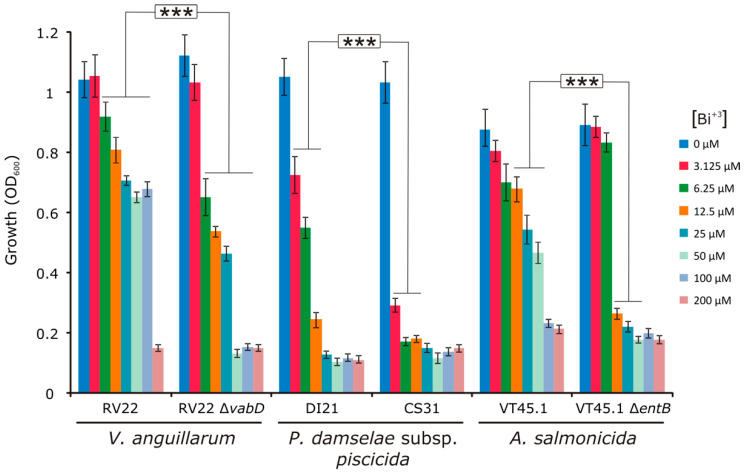
Maximum growth achieved by *V. anguillarum*, *P. damselae* subsp. *piscicida,* and *A. salmonicida* subsp. *salmonicida* siderophore-producing strains compared to their derivative siderophore deficient strains. Bacteria were grown at 25 °C for 12 h in TSB-1 containing increasing concentrations (between 0 and 200 µM) of bismuth. Asterisks (***) denote statistically significant differences (*p* < 0.001). OD = optical density.

To study whether siderophore production could affect the bismuth susceptibility of the bacteria tested, the growth achieved in presence of increasing bismuth concentrations by siderophore-producing strains was compared with the corresponding siderophore-deficient derivative mutants (Figure 2). We choose three main pathogens: *V. anguillarum* RV22, a highly pathogenic strain that produces the siderophores vanchrobactin and piscibactin [15], which are two siderophore systems widespread in *Vibrionaceae* [16]; *P. damselae* subsp. *piscicida* DI21, which produces piscibactin [17]; and *A. salmonicida* subsp. *salmonicida* VT45.1, a strain that produces the siderophores amonabactin and acinetobactin (Balado et al., 2015). The susceptibility to bismuth of these strains was compared to the susceptibility of siderophore-deficient mutants derived from each one of them: *V. anguillarum* RV22Δ*vabD*, a mutant unable to produce any siderophore; *P. damselae* subsp. *piscicida* CS31, a derivative of DI21 impaired in piscibactin synthesis [18]; and *A. salmonicida* subsp. *salmonicida* VT45.1Δ*entB*, a mutant unable to produce any siderophore [19].

The results showed that, in the three pathogens assayed, the inactivation of the siderophore systems enhanced bismuth susceptibility in vitro (Figure 2). For instance, in the range of concentrations tested, Bi(III) at 200 µM was necessary to completely inhibit the growth of *V. anguillarum* RV22, 25 µM to inhibit *P. damselae* subsp. *piscicida* DI21, and 100 µM to inhibit *A. salmonicida* VT45.1, concentrations in agreement with their respective MIC of Bi(III) (Table 1). However, the respective mutants impaired for siderophore production were inhibited by Bi(III) concentrations at least four-fold lower: 50 µM for RV22Δ*vabD*, 6.25 µM for CS31, and 12.5 µM for VT45.1Δ*entB* (Figure 2).

### 3.3. Usefulness of Deferiprone–Bismuth to Treat V. anguillarum Infections

**Figure 3:**
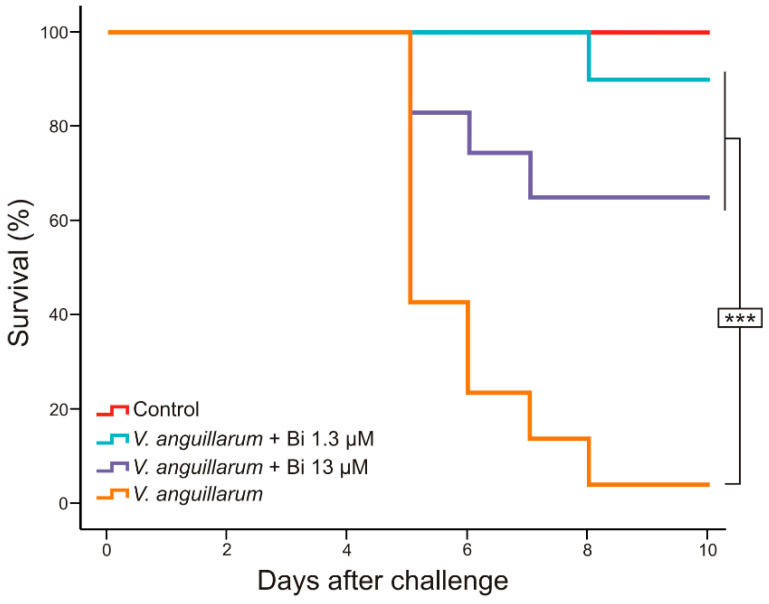
Survival rates of fish experimentally infected with *Vibrio anguillarum* RV22 and treated with 1.3 and 13 µM of Bi(III)-deferiprone. Asterisks (***) denote statistically significant differences (*p* < 0.001).

To elucidate whether the Bi(III) could be used as a chemotherapeutic to treat vibriosis in fish, an experimental challenge was made with *V. anguillarum* RV22, a highly pathogenic strain that produces vibriosis in sole (*Solea senegalensis*). The animals were inoculated with 3–5 × 10^4^ CFU of *V. anguillarum* RV22, a ten-fold dilution of a dose that initiates death events four to six days after inoculation and reaches almost 100% mortality in 10 days [15]. Four days after bacteria inoculation, two groups of fish were treated with 0.1 mL per fish of Bi(III) at either 13 μM or 1.3 µM. The survival curves are shown in Figure 3. As expected, the fish of the control group untreated with bismuth began to die five days after inoculation and the survival rate was 10% on day eight. However, in fish groups treated with bismuth a significant reduction in fish mortality was observed, both with 13 μM and with 1.3 μM Bi(III) concentrations. While survival shown by fish treated with Bi(III) 13 μM was 65%, in the group treated with Bi(III) 1.3 μM survival reached 90%. No mortality was observed in the control group whose fish were not challenged but were treated with bismuth 13 µM. In addition, control fish injected with a concentration of bismuth five times higher (65 μM) than the dose administered in the challenge showed no deaths nor apparent side effects.

## 4. Discussion

Once a bacterial disease outbreak occurs in an aquaculture facility, the only way to reduce animal mortality ratios is the use of antibacterial compounds. However, the excessive use of antibiotics increases the risk of drug resistance development in microbiota associated with fish farms and could also have adverse effects on consumers [20]. The use of alternatives to antibiotics in aquaculture, such as vaccination, probiotics, phage therapy, or essential oils are recommended to reduce the occurrence of antimicrobial residues in fish, which have consequent effects on food safety [21]. Thus, minimizing the use of antibiotics is critical for producing safe aquaculture products. In this context, the use of bismuth could be a good candidate as an antibiotic alternative to treat bacterial infections in aquaculture. Bismuth is a heavy metal environmentally safe with low toxicity for humans and animals that has been used in human medicine for more than two centuries [22].

In this work, we tested the susceptibility to Bi(III) of a collection of bacteria that can cause disease in aquatic animals. The work reported is a preliminary approach to assessing whether bismuth therapy could be applied to fight against bacterial fish diseases. Special attention was paid to three of the most relevant Gram-negative bacterial pathogens for fish: *A. salmonicida*, *P. damselae* subsp. *piscicida,* and *V. anguillarum*. Deferiprone was used to solubilize Bi(III), since it is nontoxic for animals, and it has been used for treatment of ß-thalassemia in humans [14]. Notably, the specific ligand mechanism used for bismuth stabilization as well as the overall oxidation state of the metal seems to have a significant influence on the antibacterial efficacy of deferiprone-Bi(III) [5]. The results showed that Bi(III) concentrations ranging from 13 to 130 µM were enough to significantly reduce the growth ability of most fish pathogens tested. These concentrations are comparable with those observed for other Gram-negative bacteria such as *Escherichia coli* [23]. However, a high inter- and intraspecific variability was observed.

The mechanism of action of bismuth against bacteria is not completely understood. Bismuth interfere with a range of metabolic processes such as Zn(II) and Fe(III) regulating proteins, cause cytoplasmic degradation, lead to the formation of Bi(III)-glycoproteins, bind to Fe(III)-recognition sites of transferrin and lactoferrin, and act on some metallo-enzymes including urease and alcohol dehydrogenase [22,24]. In particular, bismuth reduces ATP levels [25,26], which would be caused by targeting the F_1_ subunit of ATP synthase [27,28]. Susceptibility to bismuth among Gram-negative bacteria would be also dependent on membrane permeability and intracellular iron levels [23,29]. Our results clearly show that the ability to produce siderophores significantly enhances bismuth resistance in the three fish pathogens analyzed, *V. anguillarum*, *P. damselae* subsp. *piscicida,* and *A. salmonicida.* This observation reinforces the hypothesis that, although bismuth antibacterial activity could not be caused by intracellular iron deprivation, bismuth might act as antagonist of iron in microbial metabolism [25].

An experimental infection with *V. anguillarum* RV22 in *Solea senegalensis* showed a high reduction in the mortality ratio after treatment of fish with Bi(III) –deferiprone. Notably, the best survival rate was found when treating fish with Bi(III) at concentrations as low as 1.3 µM, a concentration 20-fold lower than the MIC observed in susceptibility tests. Notably, a bismuth concentration 10 times higher gave lower protection (Figure 3). This significant difference observed between both concentrations of bismuth tested may be explained by some type of unclear interaction effect between the pathogen, the dose of bismuth, and the host, which must be further studied. Maybe when the infection is already established, and the animal immune system is compromised, a concentration of 13 μM Bi(III) could act as antibacterial, but not only against *V. anguillarum*, but also against the animal’s microbiota, causing a dysbiosis in the individual and, consequently, its death [13,30]. However, concentrations of 65 µM Bi(III) proved to be harmless for fish. In addition to these considerations, all the results together clearly show that treatment with Bi(III) –deferiprone could be effective to minimize the infection caused by *V. anguillarum* in fish without observable undesirable side effects.

Many studies reported notable increases in antimicrobial resistance and even multiresistances in bacterial pathogens as a result of the use of antimicrobials in aquaculture [31,32,33]. Resistance genes are mostly associated with mobile genetic elements that facilitate their spread [34,35]. Bismuth therapy has been reported to show synergistic effects against bacteria when combined with antibiotics, reducing the appearance of antibiotic resistance [10,36]. Its combined use is also highly effective to combat multiresistant microorganisms [37,38,39]. Notably, since bismuth-based antimicrobial drugs serve as broad-spectrum metallo-β-lactamase inhibitors, its use could revitalize the efficacy of the existing class of beta-lactam antibiotics for which resistance has become a major issue [40]. Moreover, even with a wide use of bismuth, development of bismuth resistance in bacteria has not been reported up to now [27,41].

The results reported here suggest that bismuth therapy would be used in aquaculture systems to greatly reduce antibiotic use to treat bacterial disease outbreaks. The bismuth therapy, alone or combined with other antimicrobials, would enhance food safety and would also contribute to reduce the appearance of antibiotic resistance in pathogenic bacteria. However, further work will be necessary to find the best way to apply a bismuth-based therapeutic strategy in aquaculture facilities.

## Figures and Tables

**Table 1:** Bacterial strains used in this work and minimal inhibitory concentrations (MIC) of Bi(III) (µM) for each strain.

Bacteria	Strain ^1^	MIC of Bi (µM)
*Aeromonas hydrophila*	11	520
*A. hydrophila*	3	260
*A. salmonicida*	VT45.1	78
*A. salmonicida*	RSP74.1	13
*A. sobria*	10	130
*Lactococcus garvieae*	2	260
*L. piscium*	1	>780
*Photobacterium damselae* subsp. *piscicida*	DI21	39
*P. damselae* subsp. *piscicida*	LC14	39
*Pseudomonas anguilliseptica*	8	39
*Vibrio alginolyticus*	ACRp31.1	>780
*V. alginolyticus*	LVlenguado 27-10-09	39
*V. anguillarum*	RV22	130
*V. anguillarum*	775	130
*V. campbellii*	ATCC 25920 ^2^	>780
*V. costicola*	ACC10.2	13
*V. costicola*	ARG67.1	13
*V. diazotrophicus*	BLM05-9.1.1	>780
*V. fischeri*	IEO15.2	78
*V. fischeri*	ARG 314.1	13
*V. furnissii*	ACR159.1	>780
*V. harveyi*	AQP 15.2	130
*V. hollisae*	IEO31.2	78
*V. ichtyoenteri*	RPM799.1	39
*V. iliopiscarius*	S-SL1.2/07	>780
*V. mediterranei*	ACRp62.1	78
*V. metschnikovii*	ATCC 7708 ^2^	>780
*V. mimicus*	CECT 4218 ^2^	260
*V. nigrapulchritudo*	Nuno-3	39
*V. pelagius*	RI145.1	39
*V. scophthalmi*	ACR 318.1	78
*V. splendidus* biotype I	AZ233.1	78
*V. tapetis*	CPV7.1	39
*Yersinia ruckeri*	4	>780
*Y. ruckeri*	730	>780

^1^ All strains belong to the authors’ laboratory strain collection and have been isolated from different fish or mollusk species, except reference strains. ^2^ Reference strains obtained from ATCC and CECT culture collections.

## Data Availability

Not applicable.
